# Epidemiology of fractures and their treatment in Malawi: Results of a multicentre prospective registry study to guide orthopaedic care planning

**DOI:** 10.1371/journal.pone.0255052

**Published:** 2021-08-04

**Authors:** Alexander Thomas Schade, Foster Mbowuwa, Paul Chidothi, Peter MacPherson, Simon Matthew Graham, Claude Martin, William James Harrison, Linda Chokotho

**Affiliations:** 1 Liverpool School of Tropical Medicine, Liverpool, United Kingdom; 2 Malawi-Liverpool-Wellcome Trust Clinical Research Programme, Blantyre, Malawi; 3 AO-Alliance Foundation, Blantyre, Malawi; 4 London School of Hygiene and Tropical Medicine, Blantyre, United Kingdom; 5 Institute of Population Health Sciences, University of Liverpool, Liverpool, United Kingdom; 6 Department of Orthopaedic and Trauma Surgery, Liverpool University Teaching Hospital Trust, Liverpool, United Kingdom; 7 Countess of Chester NHS Foundation Trust, Blantyre, Malawi; 8 Department of Surgery, College of Medicine, University of Malawi, Blantyre, Malawi; Medical College of Wisconsin, UNITED STATES

## Abstract

**Importance:**

Injuries cause 30% more deaths than HIV, TB and malaria combined, and a prospective fracture care registry was established to investigate the fracture burden and treatment in Malawi to inform evidence-based improvements.

**Objective:**

To use the analysis of prospectively-collected fracture data to develop evidence-based strategies to improve fracture care in Malawi and other similar settings.

**Design:**

Multicentre prospective registry study.

**Setting:**

Two large referral centres and two district hospitals in Malawi.

**Participants:**

All patients with a fracture (confirmed by radiographs)—including patients with multiple fractures—were eligible to be included in the registry.

**Exposure:**

All fractures that presented to two urban central and two rural district hospitals in Malawi over a 3.5-year period (September 2016 to March 2020).

**Main outcome(s) and measure(s):**

Demographics, characteristics of injuries, and treatment outcomes were collected on all eligible participants.

**Results:**

Between September 2016 and March 2020, 23,734 patients were enrolled with a median age of 15 years (interquartile range: 10–35 years); 68.7% were male. The most common injuries were radius/ulna fractures (n = 8,682, 36.8%), tibia/fibula fractures (n = 4,036, 17.0%), humerus fractures (n = 3,527, 14.9%) and femoral fractures (n = 2,355, 9.9%). The majority of fractures (n = 21,729, 91.6%) were treated by orthopaedic clinical officers; 88% (20,885/2,849) of fractures were treated non-operatively, and 62.7% were treated and sent home on the same day. Open fractures (OR:53.19, CI:39.68–72.09), distal femoral fractures (OR:2.59, CI:1.78–3.78), patella (OR:10.31, CI:7.04–15.07), supracondylar humeral fractures (OR:3.10, CI:2.38–4.05), ankle fractures (OR:2.97, CI:2.26–3.92) and tibial plateau fractures (OR:2.08, CI:1.47–2.95) were more likely to be treated operatively compared to distal radius fractures.

**Conclusions and relevance:**

The current model of fracture care in Malawi is such that trained orthopaedic surgeons manage fractures operatively in urban referral centres whereas orthopaedic clinical officers mainly manage fractures non-operatively in both district and referral centres. We recommend that orthopaedic surgeons should supervise orthopaedic clinical officers to manage non operative injuries in central and district hospitals. There is need for further studies to assess the clinical and patient reported outcomes of these fracture cases, managed both operatively and non-operatively.

## Introduction

The Global Burden of Disease study estimates that in low- and middle-income countries (LMICs), injuries cause more than 220 million disability adjusted life years (DALYs) lost each year, higher than that for cancer or ischemic heart disease, or for tuberculosis, HIV and malaria combined [1]. Musculoskeletal injuries account for the majority of the disability burden from injury [2] with an estimated 130 million fractures sustained worldwide each year [3]. Fracture-related death and disability are potentially largely preventable through injury prevention schemes and accessible, good quality trauma care systems [4–6].

Malawi is a low-income country (LIC) in sub-Saharan Africa with a population of approximately 17.6 million and 84% living in rural areas [7, 8]. Fracture care is provided at district hospitals (which are typically rural), and tertiary (central) hospitals. Trained, specialized fracture care providers in Malawi include an estimated 107 non-physician orthopaedic clinical officers (OCOs) and 14 specialist orthopaedic surgeons [9]. This equates to 0.019 physicians per 1,000 people compared to the WHO standard of 2.5 physicians per 1,000 [10]. OCOs undergo 18 months of formal training on closed management of fractures and simple operative treatment, including open fracture debridement and external fixation, and serve as the primary orthopaedic care providers in district hospitals [9].

Malawi has the world’s fourth highest annual road injury mortality at 34.2 per 100,000 [11] and a high prevalence of musculoskeletal impairment [12]. However, these observations are based on extrapolation from studies done at a small number of facilities in a limited number of locations, and from surveillance systems, household surveys and facility-/hospital-level data inquiries. Attempts have been made to calculate incidences of femoral shaft fractures using hospital data and staff interviews in Malawi [13]. Without understanding the burden of injury through registries, it is difficult to inform policy and allocate resources which will achieve the greatest impact in injury prevention, treatment, and rehabilitation of injured persons. There are no sustained fracture care registries with detailed focus on skeletal injuries and their management in a LMIC [14]. The aim of this registry study was therefore to describe the burden, characteristics and treatment modalities of patients presenting to district and central hospitals with fractures using data from the registry in Malawi.

## Methods

### Study design and participants

This prospective observational registry recorded all fractures of the axial skeleton presenting to the selected hospitals over a 3.5-year period from September 2016 to March 2020. The participating hospitals were: Queen Elizabeth Central Hospital, Blantyre (QECH) and Kamuzu Central Hospital, Lilongwe (KCH), two tertiary referral hospitals with fulltime orthopaedic surgeons and OCOs; and Mangochi and Nkhata Bay District Hospitals, which are staffed by OCOs only. Data collection started at QECH and Nkhata Bay District Hospital in September 2016, at KCH in January 2017, and at Mangochi District Hospital in July 2017 to account for staff training and study coordination.

### Data collection and management

The data clerks completed the demographic details whereas the OCOs completed the clinical details. Patients who presented to either the emergency department or outpatient clinic were recruited into the registry after a diagnosis of fracture was confirmed by a clinician using an X-ray. The clinician then completed the clinical details on the registry form and then referred the patients with the registry form to the data clerk to complete the demographic details. The paper registry forms were then entered into an EPIDATA electronic database [15]. Direct electronic data capturing using Open Data Kit (ODK) [16] started from February 2019 onwards.

### Inclusion and exclusion criteria

All patients with a fracture (confirmed by radiographs)—including patients with multiple fractures—were eligible to be included in the registry. Patients with x-ray features suggestive of pathological fractures were excluded from the registry.

### Data quality assurance

At the end of every quarter, the research assistants and the principal investigator visited each participating hospital to conduct a data verification exercise. During this exercise, the number of cases recorded in the registry was compared with those recorded in the book register to determine the proportion of missed cases. Regular monitoring of the registry data, identification of errors and feedback to the team in the participating hospitals ensured improved quality of the data.

### Ethical approval

The protocol was approved by the College of Medicine Research Ethics Committee and each clinical site’s management committee. The registry collected anonymous surveillance data, hence individual consent was not required and was approved by the local research committee: P06/18/2426.

### Definitions and outcome ascertainment

Mechanism of injury was classified into road traffic accident, assault, fall, sport, animal bite, domestic violence, work related injury and other. The type of fractures were classified by orthopaedic clinical officers according to a modified AO classification including fracture pattern and laterality [17]. Management of fractures was classified as: plaster without anaesthesia; manipulation under anaesthetic and plaster; manipulation under anaesthetic and K-wiring; open fracture debridement; external fixator; skin traction; skeletal traction; intramedullary nailing; plates and screws; and other mode of management. Outcomes were classified as: treated as outpatient and sent home; admitted; referred to another facility; died; or other. Deaths were recorded during the inpatient period only.

### Statistical analysis

Data was analysed using R (The R Foundation for Statistical Computing, Vienna) [15, 18]. Descriptive statistical analysis was performed using age as a numerical variable and for the following categorical variables: sex; education; occupation; mechanism; operations; and outcome. Operations were defined as any surgical procedure that required a general, spinal or regional anaesthetic. Total number of participants and percentage were reported between district and central hospitals and compared using a Kruskal-Wallis test for non-parametric numerical data and Chi-square tests for categorical data. Where continuous data were not normally distributed, we report medians and interquartile ranges. We constructed multivariable logistic regression models to investigate associations with admission (vs. outpatient management), and a separate model for operative management (vs. non-operative management). P values below 0.05 were considered statistically significant. 95% confidence intervals were calculated. To estimate the odds of having an admission and operative management for open fractures, we excluded fractures that had a low frequency (<10) and were not long bones including clavicle, foot, hand, midshaft humerus, patella, pelvic, proximal humerus, scapula and spine.

## Results

A total of 23,733 patients with fractures were enrolled across the four hospital sites. 11,847 patients (49.9%) presented to QECH, 7,348 cases (31.0%) to KCH, 2,286 cases (9.6%) to Nkhata Bay District Hospital and 2,252 cases (9.5%) presented to Mangochi District Hospital. Overall, 63% (12,141/19,195) of patients registered at central hospitals and 47% (1,059/2,252) at district hospitals had been referred for fracture management from a lower-level health facility (Table 1). Missing data for the analysed variables was overall less than 15% and included: age = 451 (2%), education = 2,692 (13%), referral site = 1,188 (5.2%), occupation = 2,125 (9.9%), mechanism = 558 (4.2%) and open fractures = 28 (2.5%).

**Table 1:** Characteristics of fractures presenting to Malawi between 2016–2020.

	Central Hospitals (n = 19,195)	District Hospitals (n = 2,252)	P-value
Age (years, median, IQR)	15 (10–40)	15 (10–25)	<0.05
Male	13,227 (68.9%)	1,552 (67.1%)	0.629
Female	5,805 (31.0%)	698 (32.8%)	
Referred from another health facility	12,141 (63%)	1,059 (47%)	<0.05
**Education**			<0.05
No schooling/Pre-school/Vocational	3,791 (22.8%)	565 (25.9%)	
Primary	9,091 (54.7%)	1,489 (68.1%)	
Secondary	2,601 (15.7%)	110 (5.0%)	
Tertiary/University	1,132 (0.7%)	21 (<0.1%)	
**Occupation**			<0.05
Child	5,306 (31.0%)	551 (24.6%)	
Student	5,417 (31.7%)	1,068 (47.6%)	
Small business/Office worker/Self-employed	2,464 (14.4%)	156 (7.0%)	
Farmer/Labourer	2,127 (12.4%)	350 (15.6%)	
Housewife/Unemployed	905 (5.3%)	108 (4.8%)	
Other	880 (5.1%)	11 (0.5%)	
**Mechanism**			<0.05
Fall	12,594 (67.2%)	1,409 (63.3%)	
Road traffic	3,257 (17.4%)	344 (15.5%)	
Sports	928 (5.0%)	353 (15.9%)	
Assault/Domestic violence	857 (4.6%)	44 (1.0%)	
Work-related	232 (1.2%)	17 (0.8%)	
Animal bites	118 (0.6%)	24 (1.1%)	
Other	753 (4.0%)	35 (1.6%)	
**Open fractures**	1,034 (95.2%)	52 (4.8%)	<0.05
**Initial operations**			<0.05
Open fracture debridement	882 (31.9%)	50 (82.0%)	
Open reduction internal fixation	1,248 (45.1%)	2 (3%)	
IM nailing	405 (14.6%)	1 (0.2%)	
External fixation	150 (5.4%)	8 (13.1%)	
Amputations	57 (2.1%)	0	
Other	24 (0.8%)	0	
**Outcome**			<0.05
Treated and sent home	11,552 (60.2%)	1,559 (69.2%)	
Admitted	7,570 (39.4%)	687 (30.5%)	
Died	43 (0.2%)	0	
Referred	26 (0.1%)	5 (0.2%)	
Other	4 (<0.1%)	1 (<0.1%)	

Missing: age = 1,120, gender = 165, referred = 1,072, education = 2,601, occupation = 2,125, mechanism = 482, operation name = 233, outcome = 0.

### Fracture patient characteristics in central and district hospitals (Table 1)

Overall, the median age was 15 years (interquartile range [IQR]: 10–35 years). Education level was significantly higher (P<0.05) in patients treated at central hospitals, with a greater percentage of patients having university/tertiary education in central hospitals (1,132/16,615, 6.8%) compared to at the district hospitals (21/2,185, 1.0%). There were significantly more students with factures at the district hospitals (1,068/2,244, 47.6%) compared to at the central hospitals (5,417/17,099, 31.7%).

**Fig 1:**
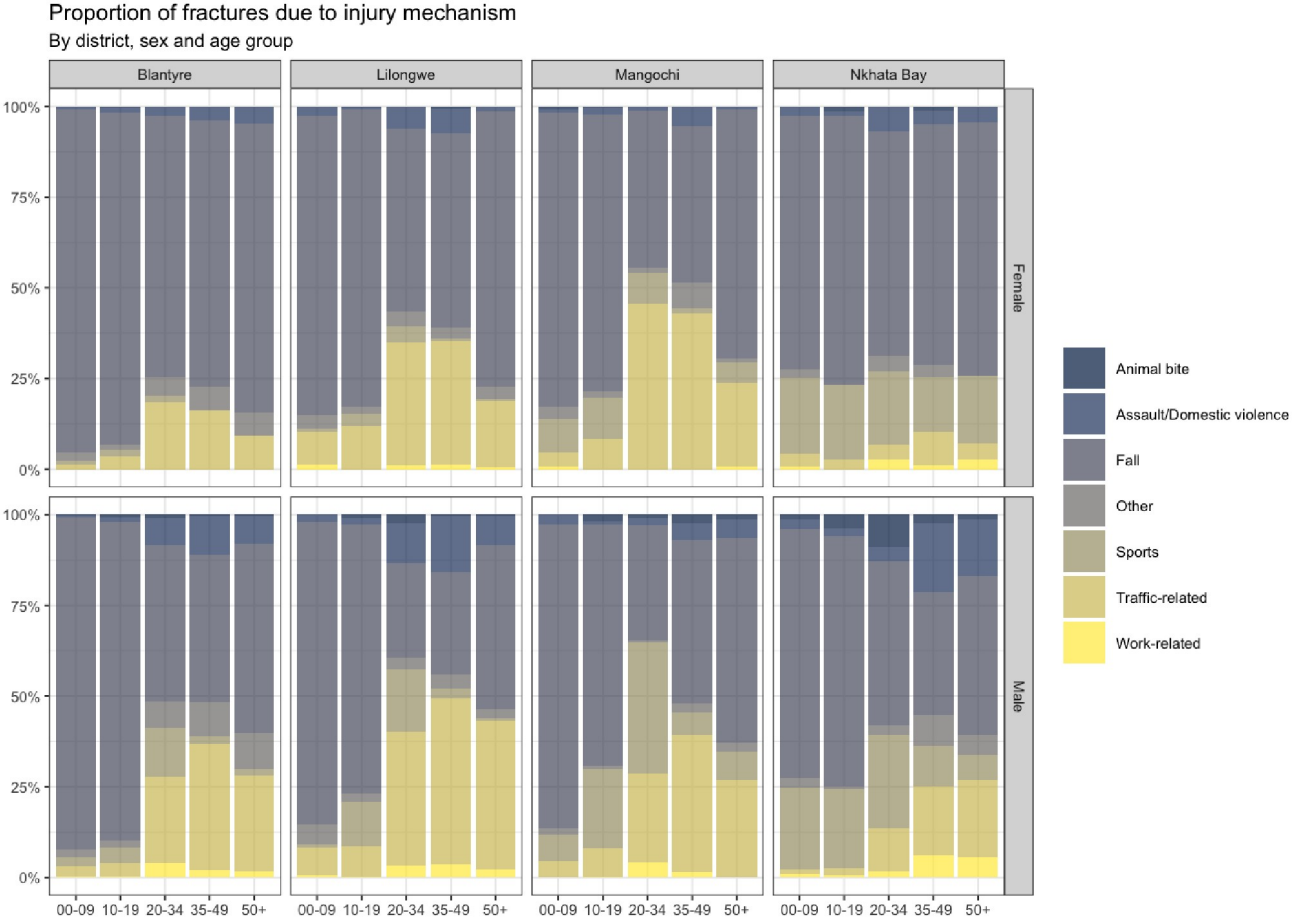
Mechanism of injury according to hospital, gender and age groups.

A significantly greater percentage (P<0.05) of patient’s fractures were due to domestic violence and assault at central hospitals (857/18,739, 4.6%) compared to district hospitals (44/2,226, 2.0%) and more animal bites (24/2,226, 1.1% vs 118/18,739, 0.6%) and sports related injuries (353/2,226, 15.8% vs 928/18,739, 5.0%) were seen in district hospitals compared to central hospitals (Table 1). Assault (266/997, 26.7% vs 114/997, 11.4%) and road traffic injuries (1,043/3,734, 27.9% vs 491/3,734 13.1%) were both more common in 20–35 year olds compared to 10–19 year olds (see Fig 1).

Eighty-two percent of fractures were treated non-operatively and there was a significantly greater percentage of operations (14.3%, n = 2,743 vs 2.9%, n = 66) performed in central hospitals compared to district hospitals (Tables 1 and 2). Orthopaedic surgeons, orthopaedic trainees and surgical trainees were only available at central hospitals. All reported deaths occurred in the central hospitals, and most commonly occurred in the over 50 year olds (45.7%, 21/46), those with femoral fractures (30.4%, 14/46) and those with a fall mechanism of injury (52.2%, 24/46).

**Table 2:** Associations with fracture hospital admission and operative management.

Variable	Admission odds ratio (95% CI)	Operative odds ratio (95% CI)
Sex		
Male	Ref	Ref
Female	0.74 (0.68–0.82)	0.72 (0.61–0.83)
Age		
0–9 years	0.46 (0.40–0.53)	0.77 (0.58–1.02)
10–19 years	Ref	Ref
20–34 years	1.17 (1.03–1.32)	1.89 (1.56–2.29)
35–49 years	1.20 (1.05–1.38)	2.28 (1.88–2.76)
50+ years	1.33 (1.15–1.55)	1.56 (1.24–1.95)
Mechanism		
Falls	Ref	Ref
Road traffic incidents	1.68 (1.49–1.90)	1.88 (1.60–2.21)
Sports	0.69 (0.58–0.83)	0.82 (0.59–1.11)
Assault/Domestic violence	1.26 (1.02–1.56)	2.00 (1.55–2.56)
Work-related	1.60 (1.09–2.31)	5.59 (3.75–8.01)
Animal bites	0.78 (0.45–1.30)	4.73 (2.78–7.79)
Study site		
Central Hospital	Ref	Ref
District Hospital	1.25 (1.11–1.41)	0.34 (0.24–0.45)
Fracture type		
Distal radius	Ref	Ref
Scapula	1.80 (0.90–3.41)	2.24 (0.86–5.95)
Clavicle	0.88 (0.68–1.15)	1.02 (0.59–1.67)
Proximal humerus	3.23 (2.60–4.01)	3.03 (2.09–4.35)
Midshaft humerus	4.95 (3.63–6.74)	3.40 (1.97–5.68)
Supracondylar humerus	5.09 (4.48–5.80)	3.10 (2.38–4.05)
Proximal radius	0.62 (0.49–0.78)	1.76 (1.24–2.47)
Midshaft radius	0.88 (0.74–1.04)	1.35 (1.00–1.82)
Hand	1.85 (1.39–2.44)	7.40 (5.27–10.37)
Pelvis	13.09 (8.50–20.71)	2.01 (1.18–3.35)
Spine	8.81 (5.45–14.64)	0.45 (0.21–0.89)
Proximal femur	21.60 (16.73–28.1)	1.72 (1.21–2.44)
Midshaft femur	23.26 (18.88–28.78)	1.50 (1.07–2.09)
Distal femur	10.21 (8.08–12.95)	2.59 (1.78–3.78)
Patella	10.68 (7.52–15.37)	10.31 (7.04–15.07)
Tibia plateau	2.38 (1.88–3.01)	2.08 (1.47–2.95)
Midshaft tibia	1.61 (1.25–2.07)	1.21 (0.80–1.82)
Distal tibia	1.18 (0.96–1.44)	1.44 (1.02–2.00)
Ankle	1.35 (1.11–1.62)	2.97 (2.26–3.92)
Foot	1.07 (0.79–1.43)	2.96 (1.98–4.37)
Fracture classification		
Closed fracture	Ref	Ref
Open fractures	29.17 (19.90–44.35)	53.19 (39.68–72.09)
Managed by		
OCO	Ref	Ref
Trainee OCO	2.05 (1.24–3.33)	0.40 (0.01–1.19)
Medical officer	8.48 (4.29–17.53)	1.28 (0.41–3.25)
Surgical trainee	123.86 (26.60–2,206.92)	6.39 (3.82–10.56)
Orthopaedic trainee	69.67 (40.28–132.98)	7.39 (5.74–9.50)
Orthopaedic consultant	114.06 (66.72–215.91)	25.11 (20.43–30.98)

(Variables adjusted for: sex, age, mechanism, fracture location, initial treating physician, hospital location and year).

The most common treatments were: 12,780 (55.9%) plaster cast without anaesthesia; 2,795 (12.2%) manipulation and plaster cast under anaesthesia; and 1,448 (6.6%) skin traction. 14,951 (62.8%) patients were discharged on the same day, 8,781 (36.7%) were admitted to hospital and 46 (0.2%) died.

### Admission and operative management (Table 2)

Regression modelling (adjusted for sex, age, mechanism, fracture location, initial treating physician, hospital location and year) indicated that women were less likely to be admitted (OR:0.74, CI:0.68–0.82) and operated on (OR:0.72, CI:0.61–0.83) compared to men (Table 2). Significantly more 35–49 year old patients were more likely to be operated on (OR:2.28, CI:1.88–2.76) compared to 10–19 year olds. Most paediatric fractures were treated non-operatively.

The majority of open fractures (1,042/1,114, 93.5%) were admitted and were more likely to be admitted (OR:29.17, CI:19.90–44.35) and operated on (OR:53.19, CI:39.68–72.09) compared to closed fractures (Table 2). Numbers of open fractures and the percentage that were initially operated remained relatively constant across the three years (2017: 270/325, 83.1%; 2018: 285/302, 94.4%; 2019: 328/387, 84.7%). Most (969/1,114, 87.0%) of the open fracture underwent operative treatment. This includes a first procedure of 943 (93%) primary debridement, 44 (4.2%) internally fixed (IM nail or open reduction internal fixation), 17 (0.2%) externally fixed and 10 (0.1%) amputation. Definitive or further procedures was not recorded as part of data collection. 52 open fractures presented to district hospitals and 50 (96.2%) received debridement, but no external or internal fixation was initially performed.

Pelvic (OR:13.09, CI:8.50–20.71), spinal (OR:8.81, CI:5.45–14.64), proximal femur (OR:21.60, CI:16.73–28.1), midshaft femur (OR:23.26, CI:18.88–28.78), distal femur (OR:10.21, CI:8.08–12.95) and patella fractures (OR:10.68, CI:7.52–15.37) were most likely to be admitted (Table 2). Proximal humerus (OR:3.03, CI:2.09–4.35), midshaft humerus (OR:3.40, CI:1.97–5.68), supracondylar humerus (OR:3.10, CI:2.38–4.05), distal femur (OR:2.59, CI:1.78–3.78), patella (OR:10.31, CI:7.04–15.07), tibia plateau (OR:2.08, CI:1.47–2.95) and ankle (OR:2.97, CI:2.26–3.92) were most likely treated operatively (Table 2).

Overall, 91.6% of fractures were treated by orthopaedic clinical officers (n = 21,729/23,734) across the four hospitals. Surgeons (orthopaedic surgeons (OR:25.1, CI:20.43–30.98), orthopaedic trainees (OR: 7.39, CI:5.74–9.50) and surgical trainees (OR:6.39, CI:3.82–10.56)) saw more of the operative cases compared to orthopaedic clinical officers (Table 2).

### Fracture characteristics by age (Table 3)

**Table 3:** Fracture types by patient characteristics.

Fracture bone	Radius (n = 8,682)			Humerus (n = 3,527)			Femur (n = 2,355)			Tibia (n = 4,036)			
Fracture location	Proximal (n = 1,266)	Midshaft (n = 2,220)	Distal (n = 5,205)	Proximal (n = 657)	Midshaft (n = 295)	Supracondylar(n = 2,808)	Proximal (n = 759)	Midshaft (n = 1,107)	Distal (n = 623)	Plateau (n = 735)	Midshaft (n = 694)	Distal (n = 1,408)	Ankle (n = 1,634)
**Sex**													
Male	888 (71%)	1,532 (70%)	3,633 (70%)	452 (69%)	207 (71%)	1,879 (67%)	463 (62%)	808 (73%)	430 (69%)	544 (75%)	495 (72%)	926 (66%)	929 (57%)
Female	367 (29%)	670 (30%)	1,547 (30%)	203 (31%)	86 (29%)	907 (33%)	287 (38%)	293 (27%)	193 (31%)	185 (25%)	193 (28%)	475 (34%)	698 (43%)
**Age**													
0–9 years	157 (13%)	297 (14%)	730 (15%)	113 (18%)	33 (12%)	534 (19%)	64 (9%)	285 (27%)	94 (16%)	56 (8%)	86 (13%)	175 (13%)	73 (5%)
10–19 years	694 (57%)	1,162 (54%)	2,662 (53%)	337 (53%)	115 (42%)	1,786 (65%)	123 (17%)	302 (28%)	170 (29%)	156 (23%)	198 (30%)	366 (28%)	259 (17%)
20–34 years	168 (14%)	321 (15%)	725 (15%)	66 (10%)	43 (16%)	196 (7%)	84 (12%)	204 (19%)	126 (22%)	158 (23%)	191 (29%)	313 (24%)	333 (22%)
35–49 years	122 (10%)	206 (10%)	514 (10%)	60 (9%)	61 (22%)	138 (5%)	135 (19%)	146 (14%)	90 (15%)	206 (30%)	134 (20%)	331 (25%)	551 (37%)
50+ years	77 (6%)	155 (7%)	209 (4%)	59 (9%)	21 (8%)	93 (3%)	297 (42%)	133 (12%)	101 (17%)	105 (15%)	54 (8%)	135 (10%)	279 (19%)
**Mechanism**													
Falls	932 (75%)	1,639 (75%)	4,139 (80%)	452 (70%)	156 (54%)	2,360 (85%)	491 (67%)	612 (57%)	346 (57%)	310 (43%)	277 (41%)	805 (59%)	975 (61%)
Road traffic incidents	99 (8%)	145 (7%)	354 (7%)	81 (12%)	74 (26%)	188 (7%)	169 (23%)	299 (28%)	162 (27%)	273 (38%)	227 (34%)	320 (23%)	367 (23%)
Sports	103 (8%)	230 (11%)	372 (7%)	66 (10%)	22 (8%)	151 (5%)	18 (2%)	37 (3%)	51 (8%)	40 (6%)	81 (12%)	107 (8%)	83 (5%)
Assault/Domestic violence	69 (6%)	105 (5%)	173 (3%)	26 (4%)	8 (3%)	50 (2%)	27 (4%)	32 (3%)	18 (3%)	50 (7%)	36 (5%)	74 (5%)	73 (5%)
Work-related	5 (<1%)	12 (<1%)	37 (<1%)	7 (1%)	5 (2%)	2 (<1%)	4 (<1%)	11 (1%)	4 (<1%)	6 (<1%)	8 (1%)	16 (1%)	16 (1%)
Animal bites	15 (1%)	42 (2%)	32 (<1%)	7 (1%)	2 (<1%)	9 (<1%)	0	2 (<1%)	2 (<1%)	5 (<1%)	13 (2%)	10 (<1%)	14 (<1%)
**Study site**													
Central Hospital	965 (87%)	1,624 (85%)	4,368 (91%)	461 (83%)	209 (75%)	2,336 (89%)	713 (98%)	886 (86%)	480 (81%)	658 (94%)	597 (91%)	1,126 (86%)	1,484 (95%)
District Hospital	147 (13%)	296 (15%)	451 (9%)	92 (17%)	69 (25%)	296 (11%)	17 (2%)	143 (14%)	110 (19%)	43 (6%)	56 (9%)	182 (14%)	84 (5%)
**Initial management**													
Operative	107 (8%)	126 (6%)	214 (4%)	69 (11%)	43 (15%)	216 (8%)	117 (15%)	114 (13%)	118 (19%)	170 (23%)	122 (18%)	195 (14%)	335 (21%)
Non-operative	11,559 (92%)	2,094 (94%)	4,991 (96%)	588 (89%)	252 (85%)	2,592 (92%)	642 (85%)	963 (87%)	505 (81%)	565 (77%)	572 (82%)	1,213 (86%)	1,299 (79%)

**Fig 2:**
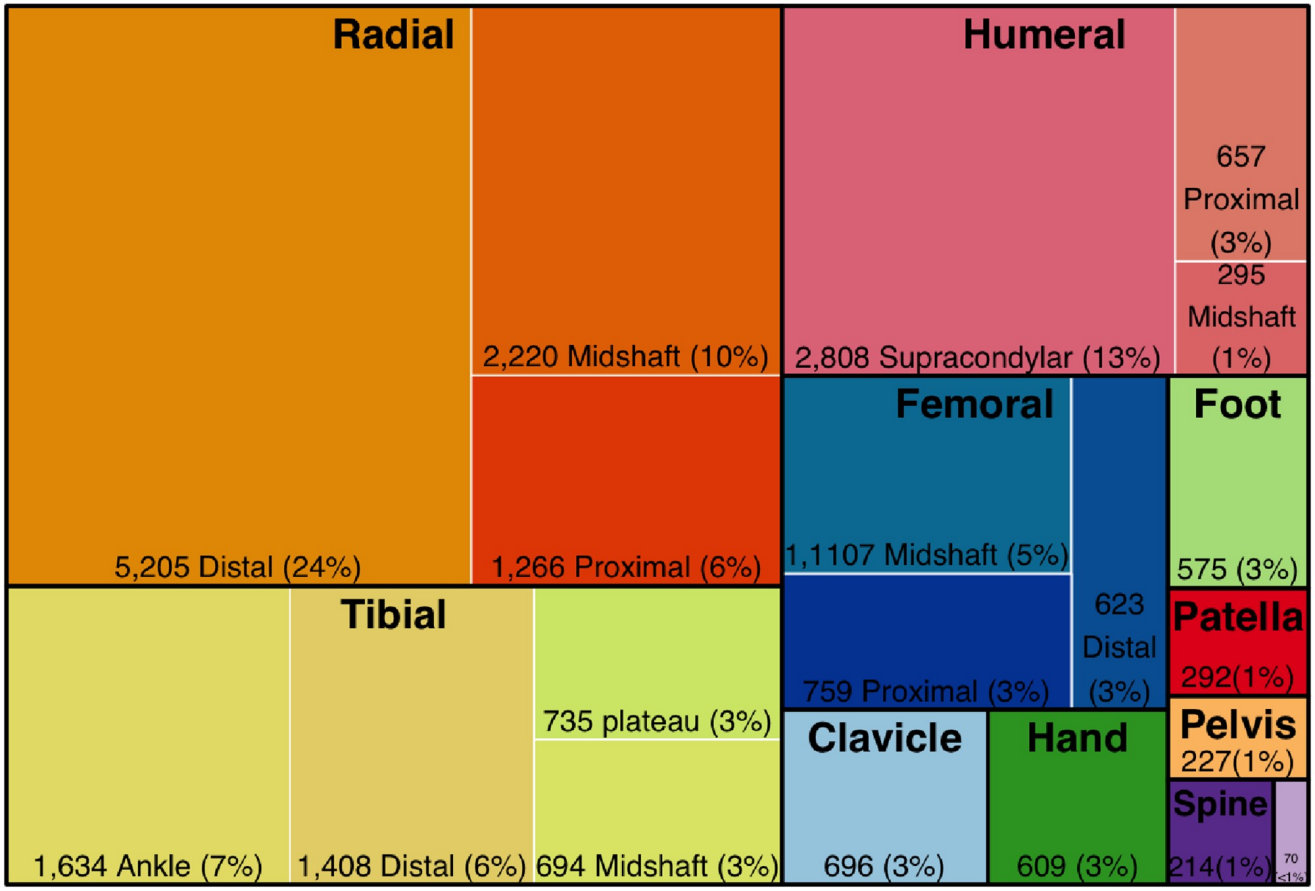
Fracture characteristics (groups are bone names, sub-groups are fracture locations, n = 23,014). 720 missing.

The most common bones fractured were the radius/ulna (8,682/23,734, 36.6%), tibia/fibula (4,036/23,734, 17.0%), humerus (3,527/23,734, 14.9%) and femur (2,355/23,734, 9.9%). Distal radius fracture were more common in the 10–19 year olds (2,662, 53%) compared to the over 50 year old (209, 4%). On the other hand, proximal femoral fractures were more common in over 50 years old (297, 42%) compared to 10–19 year olds (123, 17%). Most fractures were more common in men, but distal femur, distal tibia and ankle fractures had higher percentage in women compared to other injuries (Fig 2). The most common mechanism was falls for all fractures (15,393/23,176, 66.4%), but there was a higher percentage of injuries resulting from road traffic for midshaft femur, distal femur, tibia plateau and midshaft tibia fractures. The majority of distal radius fractures were managed as outpatients.

## Discussion

The main finding of this study is that the majority of fractures that occured in 10–19 year old individuals are due to falls and were managed non-operatively by orthopaedic clinical officers in Malawi. To our knowledge, this is the largest study describing detailed epidemiology and initial treatment of fractures presenting to hospitals in any low-income country. Overall, fractures affected a younger population than in high-income settings, likely due to the young population in Malawi (median age of 17 years) [19], and the different patterns of risk factors for fracture. However, most of the paediatric fractures were managed non-operatively with an increase in operative management in 35–49 year olds. This might be due to adults sustaining more severe fractures, such as open fractures and proximal femoral, patella and pelvic fractures, that require operative management.

With 60% of cases being referred from a health centre to a secondary or tertiary hospital, this has implications for the delay in receiving trauma care and can result in increased mortality and morbidity [20]. Most Malawian healthcare centres do not have healthcare workers with any trauma or orthopaedic training [21] or X-ray facilities and Malawian district hospitals have orthopaedic clinical officers, but very limited capacity to provide surgical services, which are restricted to central hospitals and orthopaedic surgeons [6]. Other international collaboratives have also found that open fractures are not admitted in a timely manner to hospitals in LMICs [22]. There is a need for a qualitative study to establish reasons why OCOs do not perform external fixation for open fractures in district hospitals.

Malawian district hospitals are able to provide non-operative care by orthopaedic clinical officers with supervision from orthopaedic surgeons in central hospitals. Different methods of supervision have been proposed, but in Malawi telephone consultation networks have been shown to reduce unnecessary referrals [23]. High energy fractures that require operations were treated in central hospitals. More work needs to be done to establish which injuries can be treated in district hospitals or health centres and which should be treated in central hospitals, and appropriate referral protocols need to be drafted. In HICs, the creation of major trauma networks has aided fracture care management [24, 25] and there is potential to develop a trauma network system in Malawi and similar LMICs along similar lines. The introduction of guidelines has strengthened open fracture care in the UK [25] and a recent initiative has established locally approved guidelines for the care and referral of open fractures in Malawi [26].

Most (88%, 20,885/23,734) of fractures were treated non-operatively, but it is unclear which of these fractures would have benefited from operative treatment. In high-income injuries, paediatric forearm fracture are typically treated non-operatively with good outcomes [27]. On the other hand, 85% of proximal femoral fractures (hip) are treated operatively in high-income countries as they are associated with poor quality of life and function if treated non-operatively [28–30], but our study showed only 15% of proximal femoral fractures were treated operatively. Further studies are needed to clarify the reasons behind these severe injuries not being surgically fixed in LICs. Other studies suggest these reasons could be multifactorial, including lack of basic orthopaedic equipment, OCO expertise, safe anaesthesia and sterile operating conditions [21].

More than 80% of surgical, obstetric and orthopaedic procedures could be done by associate clinicians in a practice known as “task shifting”. Malawi has 14 specialist orthopaedic surgeons and 107 OCOs. More work is required to assess clinical outcomes of fractures treated by specialist orthopaedic surgeons and orthopaedic clinical officers in Malawi. The registry data shows that orthopaedic surgeons focus on operative care, whereas OCOs focus on non-operative management. It is unclear whether this is a selection effect in that OCOs are less likely to be asked to see fractures requiring surgical intervention or is it a reflection of their decision-making skills and what the availability of resources are locally. It is important that less trained OCOs are supervised by trained orthopaedic personnel through standards and trauma networks [9].

The data from the registry does not represent all fractures in Malawi, as we included only four hospitals in Malawi [21]. In total, there are 26 district hospitals, four central hospitals and other faith based healthcare facilities that can provide orthopaedic care. It is also unlikely to represent all fractures seen in each participating facility during the study period as some cases may have been missed, especially during the night or weekends when there are fewer staff on duty. The study effect, where fewer participants are recruited at the beginning of the study, might limit the ability to conclude trends over time. The true burden of fractures is likely to be higher than our results, but we tried to mitigate this by our research team verifying the data forms onsite and comparing forms to cases in the patients’ register book every quarter. Unfortunately, there was no gold standard to assist in assessing the proportion of missing cases as during our data verification exercise, some of the book registers routinely kept by the facilities had fewer cases than our registry. Similar data quality issues in registries have also been reported in a recent systematic review [31]. This database does not provide information for timing of surgery or the follow-up treatment including complications and clinical outcomes. Further work is required to measure the morbidity and mortality from the more common fractures including the economic burden of these injuries both from the care provider perspective as well indirect costs from catastrophic loss of income. This fracture care registry only records patients that seek care in the participating hospitals in Malawi and we report much lower rates of hand (3%) and feet (3%) injuries compared to hand (25.5%) and feet (13.2%) fractures from registries in high-income countries [32]. Some of these injuries may result in long term morbidity but may not present initially to hospital.

We recommend that all countries establish systems for fracture care whereby patients can access the appropriate care for their injury in a timely fashion. In Malawi, there are only 14 orthopaedic surgeons all based in the urban areas, but 84% of the population is rural. This surgeon density distribution is not adequate to cater for the whole population but also creates access issues for the rural majority. This requires task-sharing such that many non-doctor grade workers (OCOs) who are based in district hospitals provide non-operative based care for the majority of injuries, but injuries requiring surgical care are identified and referred at the earliest opportunity to the operative-based surgeons and facilities.

The current model of fracture care in Malawi is such that trained orthopaedic surgeons manage fractures operatively in urban referral centres whereas OCOs mainly manage fractures non-operatively in both district and referral centres. We recommend that orthopaedic surgeons should supervise orthopaedic clinical officers to manage non operative injuries in central and district hospitals. There is need for further studies to assess the clinical and patient reported outcomes of these fracture cases managed both operatively and non-operatively.

## Supporting information

S1 Dataset (CSV)
